# Characterization of the complete chloroplast genome of the *Solanum tuberosum* L. cv. Atlantic (Solanaceae)

**DOI:** 10.1080/23802359.2020.1845998

**Published:** 2021-01-08

**Authors:** Jing-Ying Zhang, Jia-Yue Zhang, Yan-Fei Zhao, Shuang Li, Shan-Shan Chen, Ya-Ping Wang, Bin Mou, Hao-Ran Ma, Zhi-Jun Han, Yue Lu, Yu-Zhu Han

**Affiliations:** a College of Horticulture, Jilin Agricultural University, Changchun City, P.R. China; b Management Office of Teaching and Scientific Research Base, Jilin Agricultural University, Changchun City, P.R. China

**Keywords:** *Solanum tuberosum*, complete chloroplast genome phylogenetic analysis, Solanaceae

## Abstract

Potato (*Solanum tuberosum* L.), a species of the family Solanaceae, is the fourth most important food crop worldwide. *Solanum tuberosum* L. cv. Atlantic, a main fried special potato, has a dry matter content of 19%–23% and a starch content of 16.26% in the tuber. In order to support more molecular data for the taxony of *S. tuberosum*, the complete chloroplast (cp) genome sequence of *S. tuberosum* L. cv. Atlantic was determined using next-generation sequencing. In leaves, the chloroplast genome accounts for 5.49% of the total genome. The entire cp genome was determined to be 155,296 bp in length. It contained large single-copy (LSC) and small single-copy (SSC) regions of 85,737 and 18,373 bp, respectively, which were separated by a pair of 25,593 bp inverted repeat (IR) regions. The genome contained 132 genes, including 87 protein-coding genes, 37 tRNA genes, and eight rRNA genes. The overall GC content of the genome is 37.9%. A phylogenetic tree reconstructed by 64 chloroplast genomes reveals that *S. tuberosum* L. cv. Atlantic is most closely related to *Solanum tuberosum* L. cv. Desiree.

*Solanum tuberosum* L. (family: Solanaceae) has high nutritional value, adaptability, and large yield. It is the largest non-cereal food crop worldwide and ranked as the world's fourth most important food crop after rice, wheat, and maize (Horton and Sawyer 1985; Zhang et al. 2017). *Solanum tuberosum* L. cv. Atlantic (https://pubag.nal.usda.gov/catalog/20123), a main fried special potato cultivated variety, with bright green leaves, has a dry matter content of 19%–23% and a starch content of 16.26% in the tuber (Webb et al. 1978; Xu et al. 2019). Since the main sites of starch synthesis are amyloid and chloroplast, and the chloroplast genome contains many genes involved in starch synthesis, it is necessary to characterize the chloroplast genome of the potato Atlantic.

Healthy leaf samples were collected from a tissue culture plant (E:125.417353, N43.821995). The total genomic DNA was extracted from the fresh leaves of *S. tuberosum* L. cv. Atlantic using the DNeasy Plant Mini Kit (Qiagen, Valencia, CA, USA) and stored in College of Vegetable Science, Jilin Agricultural University (JAUDXY01). After DNA isolation, 1 μg of purified DNA was fragmented and used to construct short-insert libraries (insert size ∼350 bp) according to the manufacturer’s instructions (BGISEQ) detailed in the previous literature (Huang et al. 2017). Then DNA librarie were sequenced by Hefei Bio&Data Biotechnologies Inc. (Hefei, China) on the BGISEQ-500 platform with PE150 read lengths. The filtered reads were assembled using the program NOVOPlasty Version 3.8.3 (Dierckxsens et al. 2017). The cp-genome was annotated with the DOGMA (Wyman et al. 2004) and tRNAscan (Schattner et al. 2005).

In the eaves of *Solanum tuberosum* L. cv. Atlantic, the chloroplast genome accounts for 5.49% of the total genome. Such a high proportion of the chloroplast genome may have contributed to its bright green leaves and high starch production. The chloroplast genome was determined to comprise double stranded, circular DNA of 155,296 bp containing two inverted repeat (IR) regions of 25,593 bp each, separated by large single-copy (LSC) and small single-copy (SSC) regions of 85,737 and 18,373 bp, respectively (Genome Warehouse acc. no. GWHAORW01000000). The genome contained 132 total genes, including 87 protein-coding genes, 37 tRNA genes, and eight rRNA genes. Seven protein-coding genes, six tRNA genes and four rRNA genes were duplicated in IR regions. nineteen genes contained two exons and four genes (clpP and ycf3 and two rps12) contained three exons. The overall GC content of *S. tuberosum* L. cv. Atlantic cp genome is 37.9% and the corresponding values in LSC, SSC and IR regions are 36.0, 32.1 and 43.1%, respectively. Heteroplasmy testing showed that there are about 141 low-frequency SNP sites with minor allele frequency (MAF) ≥0.03 and ≥5× reads coverage in the chloroplast genome of potato Atlantic. Most of these SNPs are located between ycf15 and trnL-CAA.

To investigate its taxonomic status, a maximum likelihood (ML) was reconstructed based on whole chloroplast genomes from 63 *Solanum* plants and one outgroup plants (*Eucommia ulmoides*) (Figure 1) by Mafft version 1.4 and FastTree version 2.1.10 (Price 2010). The ML phylogenetic tree shows that *Solanum tuberosum* L. cv. Atlantic is most closely related to *Solanum tuberosum* L. cv. Desiree. with bootstrap support values of 100%. Chloroplast genome of *S. tuberosum* L. cv. Atlantic adds valuable information for understanding the phylogenetic position of *S. tuberosum* in the genus *Solanum*.

**Figure 1.**
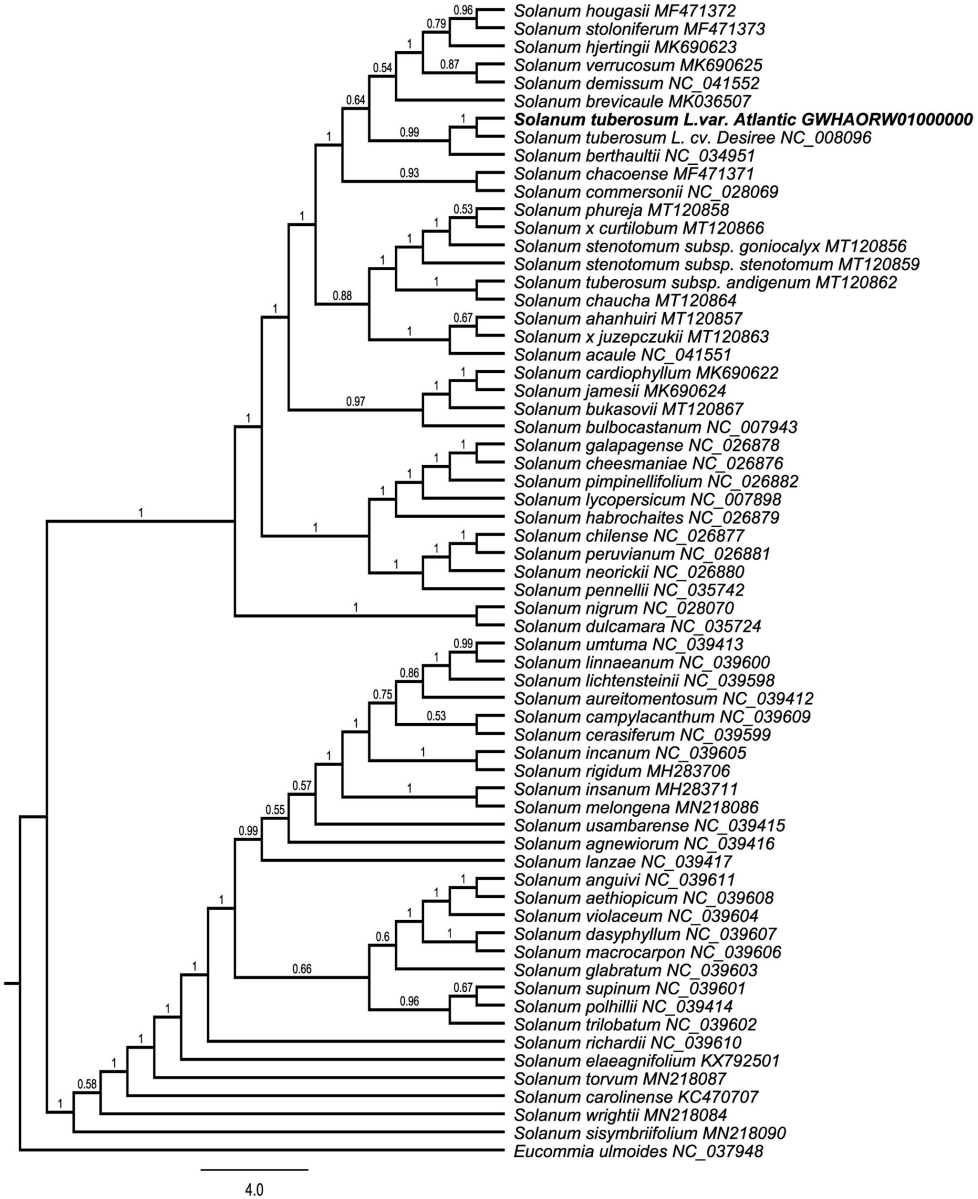
Maximum-likelihood phylogenetic tree based on whole chloroplast genomes from 63 *Solanum* plants and one outgroup plant (*Eucommia ulmoides*) and the support values are shown at the branches.

## Data Availability

The complete chloroplast genome sequence of *Solanum tuberosum* L. cv. Atlantic is deposited in the Genome Warehouse (https://bigd.big.ac.cn/gwh/Assembly/10360/show) database under the accession number GWHAORW01000000. The raw sequencing data is deposited in the Genome Sequence Archive database (https://bigd.big.ac.cn/gsa/browse/CRA003215/CRR180692) under the accession number CRR180692.
